# From Rarity to Recognition: Infantile Botulism and the Broad Spectrum of Differential Diagnoses

**DOI:** 10.1155/2024/4647591

**Published:** 2024-02-26

**Authors:** Matthew C. Authement, Brandon M. Jones, Robert J. Kahoud, Elizabeth H. Ristagno

**Affiliations:** ^1^Pediatric Hospital Medicine, Department of Pediatrics, Children's Hospital and Medical Center, Omaha, Nebraska, USA; ^2^Division of Child and Adolescent Neurology, Mayo Clinic, Rochester, Minnesota, USA; ^3^Division of Pediatric Critical Care Medicine, Mayo Clinic, Rochester, Minnesota, USA; ^4^Division of Pediatric Infectious Disease, Mayo Clinic, Rochester, Minnesota, USA

## Abstract

This case illustrates a 5-week-old girl who presented with decreased activity, decreased feeds, poor suck, weak cry, lethargy, hypotonia, and areflexia. The child was found to have infant botulism. The case demonstrates the importance of a full history and broad differential in an ill-appearing infant. The differential for an ill-appearing infant should always include infectious etiologies and may include metabolic disorders, congenital anomalies, nonaccidental trauma, neurologic disorders, and endocrine disorders. The broad differential diagnosis may make rapid diagnosis and treatment for infantile botulism a challenge.

## 1. Introduction

Infantile botulism arises from ingestion of *Clostridium botulinum* spores which colonize the gastrointestinal tract and release the botulinum toxin. *Clostridium botulinum* incubates 10–30 days prior to symptoms [1]. The botulinum toxin irreversibly binds to glycoprotein structures on cholinergic nerve terminals leading to an intracellular blockade of acetylcholine secretion [2]. Since the clinical diagnosis of infantile botulism is imperative in order to initiate prompt treatment with BabyBIG, treatment should not be delayed for confirmatory testing. Environmental exposures through spores in soil and dust contribute to the majority of cases of infantile botulism compared to honey exposure in the United States [3].

## 2. Case

A 5-week-old girl born at 37 weeks gestation by normal spontaneous vaginal delivery with history notable for oral thrush for the past 2 weeks that has not responded to nystatin, poor weight gain since birth, and microcephaly presented to the emergency department with 3 days of decreased activity, decreased feeds, poor suck, weak cry, and lethargy. The child had no history of fever, vomiting, diarrhea, cough, congestion, increased work of breathing, or sick contacts. The patient's last bowel movement was 2 days prior to presentation. Newborn screen showed alpha thalassemia trait. She was fed with breast milk and formula.

Initial vitals included a temperature of 36.3°C, heart rate of 168 beats/min, oxygen saturation of 60% on room air, and respiratory rate of 32 breaths/min. Examination showed an ill-appearing, lethargic infant with a nearly absent cry. Her anterior fontanelle was sunken and head circumference was 34.5 cm (1^st^ percentile). Pupils were reactive. There was no ophthalmoparesis. Mucous membranes were dry. She had weak pulses with a capillary refill >5 seconds, poor skin turgor, and mottling. Auscultation revealed diffuse coarse breath sounds and crackles without an increased work of breathing. Her abdomen was soft without distension, hepatomegaly, or tenderness. External genitalia appeared normal and not virilized. There were appendicular and axial hypotonia and marked head lag. Patellar, biceps, and brachioradialis reflexes were absent. Suck reflex was present but weak. There was a gag reflex.

Serum glucose was 268 mg/dl, pH was 7.27, and urine glucose was 250 mg/dl. Cerebrospinal fluid (CSF) contained 125 total nucleated cells/mcL (94% neutrophils, 3% lymphocytes, and no blasts), 287,000 erythrocytes/mcL, glucose was 74 mg/dL, protein was 796 mg/dL, and CSF multiplex PCR (which tests for *Escherichia coli* K1,*Haemophilus influenzae*, *Listeria monocytogenes*, *Neisseria meningitidis*, *Streptococcus agalactiae*, *Streptococcus pneumoniae,* cytomegalovirus, enterovirus, herpes simplex virus 1 and 2, human herpesvirus 6, human parechovirus, varicella zoster virus, and *Cryptococcus neoformans/gattii;* BioFire FilmArray, Salt Lake City, UT) and CSF HSV PCR were negative. Serum glucose at the time of lumbar puncture was 91 mg/dL. Nasopharyngeal SARS-CoV-2 RNA PCR was undetected. Complete blood count, basic metabolic panel, urine drug screen, lactate, ammonia, and CRP were normal. Chest X-ray showed right basilar atelectasis. Head CT without contrast was normal. Intraosseous access was obtained for fluid administration and antibiotics in the emergency department. She was started on continuous positive airway pressure and saturations improved. After transfer to the pediatric intensive care unit, further imaging, procedures, and laboratory testing were performed.

During hospitalization, the infant required intubation on day 2 of hospitalization due to increasing apneic events. Due to suspicion for infantile botulism, human-derived botulism immune globulin (BabyBIG) was given on the fourth day of admission.

**Figure 1:**
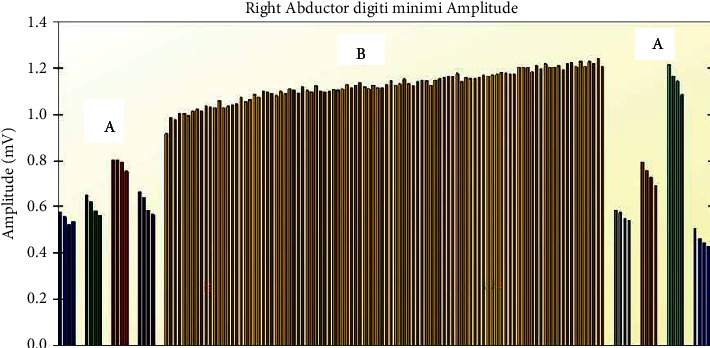
EMG of right abductor digiti minimi. A represents low amplitude baseline action potentials which decrease with four repetitive stimuli at 2 Hz. B represents facilitation given the increasing amplitude with faster rates of repetitive stimulation to represent exercise.

Given the infant's lack of stooling, stool test for direct toxin analysis and culture were obtained using tap water enema and sent to the state health department. This was positive for botulinum toxin type A by mouse bioassay by the fifth day of hospitalization. Prior to the results of the stool studies, electromyography was performed as shown in Figure 1. This revealed low compound muscle action potential amplitudes at baseline that decreased with four repetitive stimuli at a frequency of 2 Hz and faster rates of repetitive stimulation representing facilitation, supporting the clinical diagnosis of botulism. Final stool culture results were available by the tenth day of hospitalization.

After receiving BabyBIG on day 4, she had gradual improvement in tone. She was extubated within days of receiving BabyBIG but required frequent suctioning for secretions. She was discharged from the hospital on nasogastric tube feeds which were required for approximately 3 weeks after BabyBIG.

## 3. Discussion

The differential diagnosis for the lethargic infant is broad. Given the ill appearance of this infant, infectious causes such as sepsis and meningitis must be considered. Absence of fever does not preclude infection and this patient was initially slightly hypothermic at 36.3°C. Ill-appearing infants should undergo a full workup, especially given this patient's age of 5 weeks.

The combination of areflexia, hypotonia, weakness, dysphagia, and constipation suggests disorders that impair both motor unit and autonomic function. These include disorders of neuromuscular junction, polyradiculopathies, such as Guillain–Barre syndrome, and anterior horn cell diseases, including acute flaccid myelitis and spinal muscular atrophy [2, 4]. In the absence of anatomic abnormality, inability to manage secretions is indicative of bulbar dysfunction with or without altered mental status [2]. EEG may be required to rule out subclinical seizures if the child has an impaired or fluctuating level of consciousness. Encephalopathy secondary to electrolyte abnormalities such as hypoglycemia, hypocalcemia, and hyponatremia can lead to lethargy in infants.

Congenital adrenal hyperplasia and hypothyroidism may present as lethargy in infants. In this patient, the absence of virilization with normal potassium and bicarbonate speaks against this diagnosis. Newborn screening for 17-hydroxyprogesterone detects up to 90% of cases of congenital adrenal hyperplasia, since 21-hydroxylase deficiency is the most common cause of congenital adrenal hyperplasia [5]. Primary hypothyroidism is usually identified by newborn screening.

Congenital heart disease and arrhythmias, specifically supraventricular tachycardia, can present as poor feeding with hypoxia and failure to thrive and are investigated with electrocardiography and echocardiography.

Other diagnoses to consider in this case include nonaccidental trauma, inborn errors of metabolism, electrolyte abnormalities, ingestion, and other genetic causes [4]. Genetic disorders may have chronic, acute, or acute on chronic presentations. This infant had poor weight gain since birth and was microcephalic. This prompted chromosomal testing including chromosomal microarray and mitochondrial DNA full genome analysis which were normal.

Infantile botulism is characterized by a descending flaccid paralysis of the motor and autonomic nerves [6]. Patients with infantile botulism typically present at a median age of 16 weeks ranging from 1 to 60 weeks [7]. In infants, this classically presents with poor feeding, weak cry, constipation, and proximal greater than distal hypotonia [8]. Constipation accompanies infantile botulism in 95% of patients. Botulinum toxin does not cross the blood-brain barrier [6]. Encephalopathy, if present, would be secondary to significant electrolyte or cardiorespiratory disturbances. Only about 5–15% of cases can be connected to honey ingestion, but the majority occur from exposure to spores from dust and soil [3, 6]. Further history from this patient's family was negative for honey or syrup ingestions. There was no known construction near the family's home that could have led to an increase in spores from soil; however, exposure from spores from dust or soil was most likely the cause for this patient given no other source.

Treatment for infantile botulism focuses mainly on supportive care and prevention of worsening neuromuscular blockade [8]. Supportive care consists of elevating the head of the bed to 20° with a neck roll to maintain airway protection [8]. Constipation should be addressed with enemas, early feeding, stool softeners, and suppositories [8]. Care should be taken with diaper changes as infants will excrete botulism toxin in feces for weeks to months. Those with cuts should wear gloves to prevent wound botulism and dirty diapers should be disposed of where animals and children cannot access to prevent foodborne botulism [6].

BabyBIG should be given to patients based on clinical suspicion. Clinicians can utilize the Centers for Disease Control and Prevention flowchart to assist with diagnostic testing and treatment [9]. BabyBIG is FDA approved for infantile botulism and consists of pooled immunoglobulin from adults administered botulinum toxin. BabyBIG treats only infantile botulism due to type A and B toxins. The FDA has approved BabyBIG for patients less than one year of age. This immunoglobulin provides six months of protection from botulism toxin. BabyBIG has been shown to decrease the average length of hospital stay, ICU stay, mechanical ventilation, and tube feeding [10]. BabyBIG enhances elimination of the botulism toxin from blood which prevents further binding of toxin at the presynaptic nerve terminal. Recovery depends on regeneration of presynaptic terminal function [11]. For patients greater than one year of age, equine serum heptavalent botulism antitoxin should be utilized. This has antibodies for botulism toxins A, B, C, D, E, F, and G [12]. Equine serum botulism antitoxin is FDA approved but has a higher side effect profile which makes it a second-line therapy in the setting of patients less than one year of age [12].

In addition, antibiotics can lead to worse outcomes, especially if aminoglycosides are used. Aminoglycosides lyse *Clostridium botulinum* leading to increased toxin release and potentiate neuromuscular weakness [13].

## 4. Conclusion

Ill appearance in an infant with lethargy, hypotonia, and poor weight gain should elicit a broad differential encompassing infectious, metabolic, genetic, and neurologic diseases. If initial testing is unrevealing, infantile botulism can be considered, especially if the infant is constipated and hypotonic. Lack of exposure to honey should not falsely reassure providers when considering infantile botulism.
